# Development of a markerless tumor-tracking algorithm using prior four-dimensional cone-beam computed tomography

**DOI:** 10.1093/jrr/rry085

**Published:** 2018-11-08

**Authors:** Ritu Bhusal Chhatkuli, Kazuyuki Demachi, Mitsuru Uesaka, Keiichi Nakagawa, Akihiro Haga

**Affiliations:** 1 Department of Nuclear Engineering and Management, School of Engineering, The University of Tokyo, 7-3-1 Hongo, Bunkyo-ku, Tokyo, Japan; 2 Department of Radiology, The University of Tokyo hospital, 7-3-1 Hongo, Bunkyo-ku, Tokyo, Japan; 3 Graduate School of Biomedical Sciences, Tokushima University, 3-18-15, Kuramoto-cho, Tokushima, Japan

**Keywords:** tumor tracking, markerless, 4D-CBCT, phase recognition

## Abstract

Respiratory motion management is a huge challenge in radiation therapy. Respiratory motion induces temporal anatomic changes that distort the tumor volume and its position. In this study, a markerless tumor-tracking algorithm was investigated by performing phase recognition during stereotactic body radiation therapy (SBRT) using four-dimensional cone-beam computer tomography (4D-CBCT) obtained at patient registration, and in-treatment cone-beam projection images. The data for 20 treatment sessions (five lung cancer patients) were selected for this study. Three of the patients were treated with conventional flattening filter (FF) beams, and the other two were treated with flattening filter-free (FFF) beams. Prior to treatment, 4D-CBCT was acquired to create the template projection images for 10 phases. In-treatment images were obtained at near real time during treatment. Template-based phase recognition was performed for 4D-CBCT re-projected templates using prior 4D-CBCT based phase recognition algorithm and was compared with the results generated by the Amsterdam Shroud (AS) technique. Visual verification technique was used for the verification of the phase recognition and AS technique at certain tumor-visible angles. Offline template matching analysis using the cross-correlation indicated that phase recognition performed using the prior 4D-CBCT and visual verification matched up to 97.5% in the case of FFF, and 95% in the case of FF, whereas the AS technique matched 83.5% with visual verification for FFF and 93% for FF. Markerless tumor tracking based on phase recognition using prior 4D-CBCT has been developed successfully. This is the first study that reports on the use of prior 4D-CBCT based on normalized cross-correlation technique for phase recognition.

## INTRODUCTION

Radiation therapy is a highly adapted procedure for both palliative and curative treatment of cancer. The technology has rapidly progressed in the last two decades to improve its precision and effectiveness. It is largely the use of image-guided radiation therapy (IGRT) that has taken it to a high level. On the other hand, intrafraction motion caused by respiratory motion is a major issue for modern-day IGRT [1]. The uncertainties caused by respiratory motion are taken into account by expanding the treatment margin, ultimately leading to the irradiation of healthy surrounding tissues.

Various motion management approaches have been investigated, including breath holding during the treatment, and respiratory gating based on the tracking of implanted fiducial markers. These approaches add external burden for the patient. Deriving tumor positions from the external surrogates has been implemented clinically for gated lung cancer therapy [2] However, such techniques are highly dependent on correlation between the target motion and the external surrogate. On the other hand, a non-real-time markerless tracking approach has been successfully applied for tracking tumor motion using a cone-beam computed tomography (CBCT) device [3]. Real-time registration using a kilovolt–megavolt image has been reported for motion tracking, though the technique has lacked synchronization between the kilovolt and megavolt image pairs [4].

In our hospital, a kilovolt imaging system (capable of radiography, fluoroscopy and CBCT) integrated with a medical linear accelerator has been implemented, and a clinical workflow using 4D-CBCT for stereotactic volumetric-modulated arc therapy (VMAT) for lung treatment has been established as an early stage of 4D-CBCT development [5, 6]. Two sets of 4D-CBCT (i.e. prior and during the treatment) are acquired as part of the treatment process. Although 4DCT is widely used for the tracking of moving tumors, 4D-CBCT is considered more useful because it is taken just prior to treatment for patient registration and positioning. Using 4D-CBCT, a respiratory-phasecorrelated lung tumor volume can be visualized just before the treatment starts [5, 7–9]. In addition, in-treatment 4D-CBCT is acquired during the treatment in order to verify the tumor position; the treatment is verified by comparing it with the planning CT obtained a week before the treatment.

This study was inspired by our previous work in which we reported a feasibility study on tracking moving tumors using dynamic image prediction [10]. In this study, we report a more practical approach involving collection of the available imaging datasets. We used these data for the development of a prior image–based phase recognition (PIPR) algorithm for tracking moving tumors. We used the CBCT obtained just prior to the treatment, and the in-treatment images were obtained during the treatment.

## MATERIALS AND METHODS

**Fig. 1.**
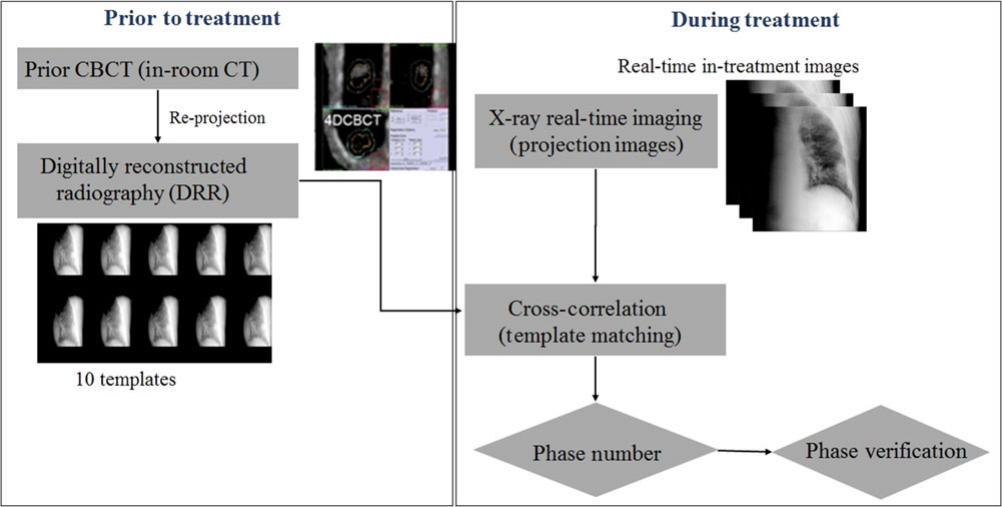
PIPR algorithm for phase-based tracking, prior to treatment (left) and during the treatment (right).

The data for 20 treatment sessions, including that of five patients with lung cancer (three treated with flattening filter beams, and two treated with flattening filter-free beams), were selected for this study. These data were acquired following the ethical guidelines in our hospital, with written consent obtained from each patient before the initiation of the treatment. All patients were treated using the Elekta Synergy Linac, consisting a conventional 120 kV X-ray source and a silicon detector, both mounted on the gantry. The details of the PIPR algorithm are illustrated in Fig. 1.

Though this study was performed offline, the algorithm itself can be divided into a prior-to-treatment section and a during-treatment section to demonstrate its application to the treatment in real time. Prior to the treatment, the 4D-CBCT was obtained and was re-projected to 10 templates, divided according to the phase of the respiratory cycle. These 10 templates were used for matching with the in-treatment images at corresponding angle to determine the correct phase. Once the phase was recognized, it was verified visually by comparing it with the phase obtained using the AS technique, and the phase obtained by our PIPR algorithm. (Only limited tumor-visible angle case).

### 4D-CBCT acquisition

Acquisition of prior 4D-CBCT just before the treatment is a regular part of treatment in our hospital, and is used for patient set-up and registration. Although 4D-CBCT acquisition is currently provided by Elekta, it can also be acquired by in-house software using the Feldkamp, Davis and Kress (FDK) algorithm [5, 11, 12]. The 4D-CBCT images can be reconstructed by classifying the acquired projection images into respiratory phases divided into several bins.

**Fig. 2.**
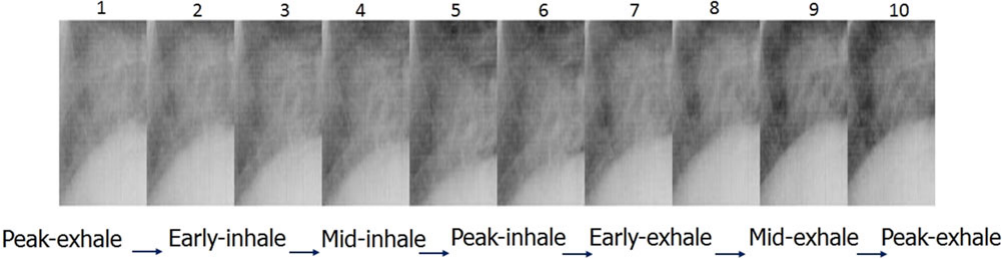
Schematic example of respiratory cycle divided into 10 phases.

**Fig. 3.**
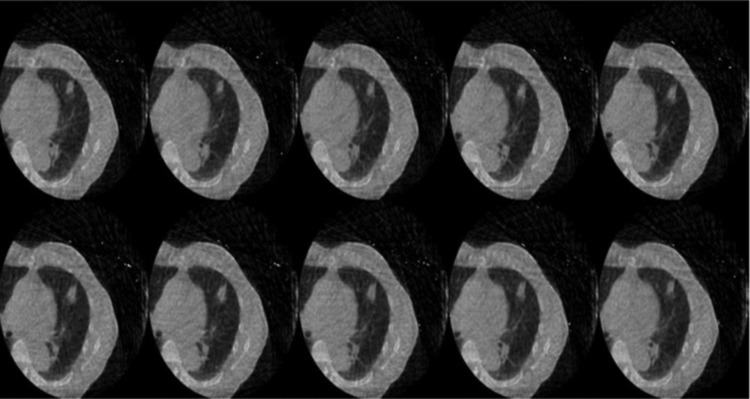
Reconstructed 4D-CBCT (10 phases) for patient data.

Figure 2 shows the classification of phases according to the region of interest, which is approximated tentatively for the anticipated motion range of the tumor. The cycle itself is classified into 10 phases, from peak exhale to early, mid and peak inhale, and back to early, mid and peak exhale. The reconstructed 4D-CBCT for 10 phases for one of the patient’s data is shown in Fig. 3. After the acquisition of reconstructed 4D-CBCT, the next step is to use this reconstructed 4D-CBCT to re-project the images for the 10 phases for each angle using interpolated angular data obtained from the prior angle information. These re-projected images were used as template-projected images.

### Re-projection

**Fig. 4.**
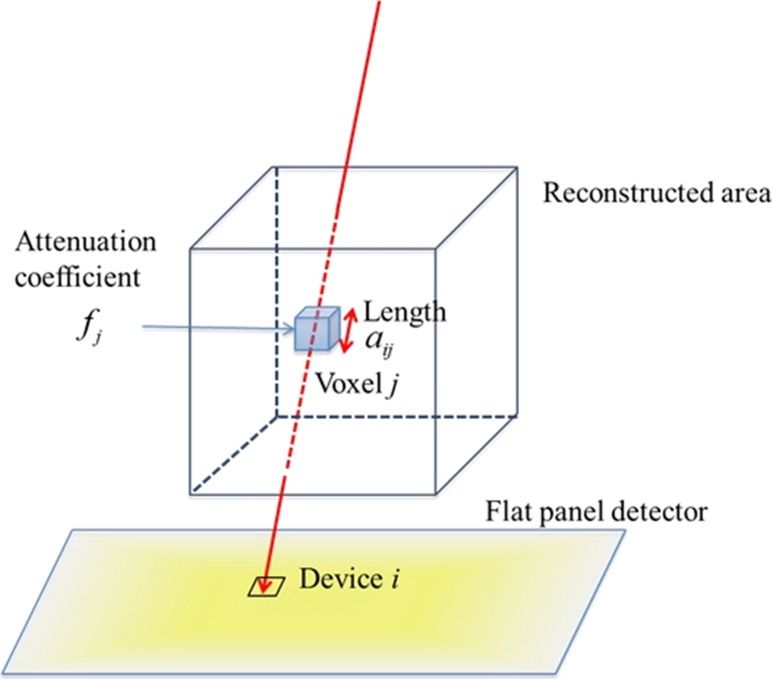
Re-projecting geometry for the acquisition of template-projected images.

This part is the second step in the phase recognition algorithm that is performed prior to the treatment. In this study, the 4D-CBCT images were re-projected in one-degree intervals (−180° to +180°) using a naive re-projection algorithm. In Fig. 4, we can see the relative locations of the X-ray pass, the reconstructed area, and the flat panel detector. Here, the re-projection is performed by calculating the attenuation coefficient along the red line:

(1) $$P_{i}=P_{o}e^{-\sum_{j}a_{ij}f_{j}},$$

where, *f*_*i*_ is the attenuation coefficient in voxel *j* and was estimated from the CT intensity values, and *a*_*ij*_ is the length passing through the corresponding voxel. The X-ray intensity in source *P*_*o*_ is attenuated to *P*_*i*_ in the detector device pixel *i*.

This was done for all of the 10 phases in the 4D-CBCT images for the whole 360 degrees.

### In-treatment image acquisition and template matching for phase recognition

In the previous sections, we have described the preparation of the template-projected images. As mentioned earlier, since this study was conducted offline, the in-treatment images were obtained along with the prior images. Images are acquired at the rate of 0.18 s per frame from the X-ray volume imaging (XVI) system. While performing online, the in-treatment images can be acquired in near real time (~0.15 s) from the projection streaming client provided by Elekta itself. In this section, we show how to perform a matching between the template-projected images (region of interest) and the in-treatment images (region of interest) at each angle. The matching was performed using a cross-correlation technique that is widely used for evaluating the similarity between the two images [13–15]. Once the in-treatment images were obtained in near real time, each image at each angle underwent matching with the 10 phase templates. Normalized cross-correlation was used for matching to evaluate the phases. The cross-correlation coefficient was calculated using the following equation:

(2) $$Cor=\frac{\sum_{m}\sum_{n}\left(A_{mn}-A_{av}\right)\left(B_{mn}-B_{av}\right)}{\sqrt{\left(\sum_{m}\sum_{n}{\left(A_{mn}-A_{av}\right)}^{2}\right)\left(\sum_{m}\sum_{n}{\left(B_{mn}-B_{av}\right)}^{2}\right)}},$$

where *A*_*mn*_ is the in-treatment image, *A*_*av*_ is the average and, similarly, *B*_*mn*_ is the template image; *B*_*av*_ is the average and *mn* is the number of pixels in the image.

In this research, the result obtained (i.e. the output phase result obtained by using the PIPR algorithm, using normalized cross-correlation) was verified using visual verification. The criteria for determining the phase match is defined as follows:

(i) Two-phase criterion: where the obtained phase number is equal to or within two phases of the actual phase. Then, the phase number obtained and the phase number visually verified are matched.

Phases are matched if *P*_*obt*_ and *P*_*vv*_ satisfy the following equation:

(3) $$-2\leq \left|P_{obt}-P_{vv}\right|\leq 2,$$

where *P*_*obt*_ is the phase number obtained using our PIPR algorithm, and *P*_*vv*_ is the phase number obtained by visual verification.

(ii) Relative phase criterion: in this criterion, we assume that respiratory motion is cyclic in most cases, and that the phases move in order (i.e. 1-2-3-4-5-6-7-8-9-10-1-2-3-4 etc.). There is little possibility that the tumor will skip the order and move to a different phase number unless there is any sudden abnormal motion.

We mark the phases as matched if the tumor is in a similar position and shape (as in mid-exhale or mid-inhale phase) and the difference in the value of cross-correlation coefficient is <0.01 shown as,

(4) $$\left|CC_{vv}-CC_{op}\right|\leq 0.01,$$

where, *CC*_*vv*_ and *CC*_*op*_ are the cross-correlation coefficient values for the visually verified phase and the phase obtained using our algorithm, respectively.

## RESULTS

**Fig. 5.**
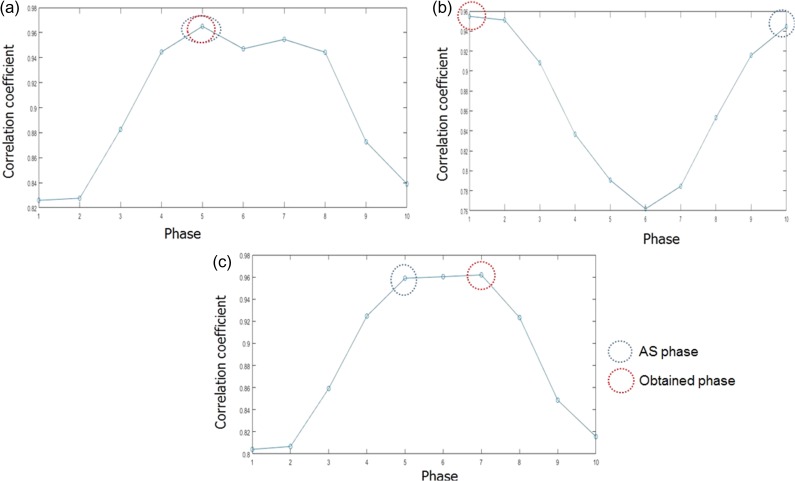
Template matching results using cross-correlation for random angles: (a) 90°, (b) 91°, and (c) 106°.

The template-matching results for a few randomly selected angles are shown in Fig. 5. In Fig. 5a, we can see that the AS phase (i.e. the phase obtained by the Amsterdam shroud technique) was equal to the phase obtained by our phase recognition algorithm. In Fig. 5b, it can be seen that the AS phase was Phase 10 and the obtained phase was Phase 1. Both the phases are at the peak-exhale phase and are hence considered the same. In Fig. 5c, we can see that the actual phase and the predicted phase were within two phase differences. In this case, the tumor was in the mid-inhale and the early-exhale phases respectively in which the tumor shape and position are almost similar. The calculation time for the cross-correlation algorithm was <100 ms in each case.

### Visual verification

After performing phase recognition using template matching at each angle, we verified the phases obtained from the PIPR algorithm and the AS technique by comparing them with the phases recognized visually. Due to the limitation of the view of the tumor by the limited number of images, comparison with visual verification was limited to images obtained from certain angles only.

**Fig. 6.**
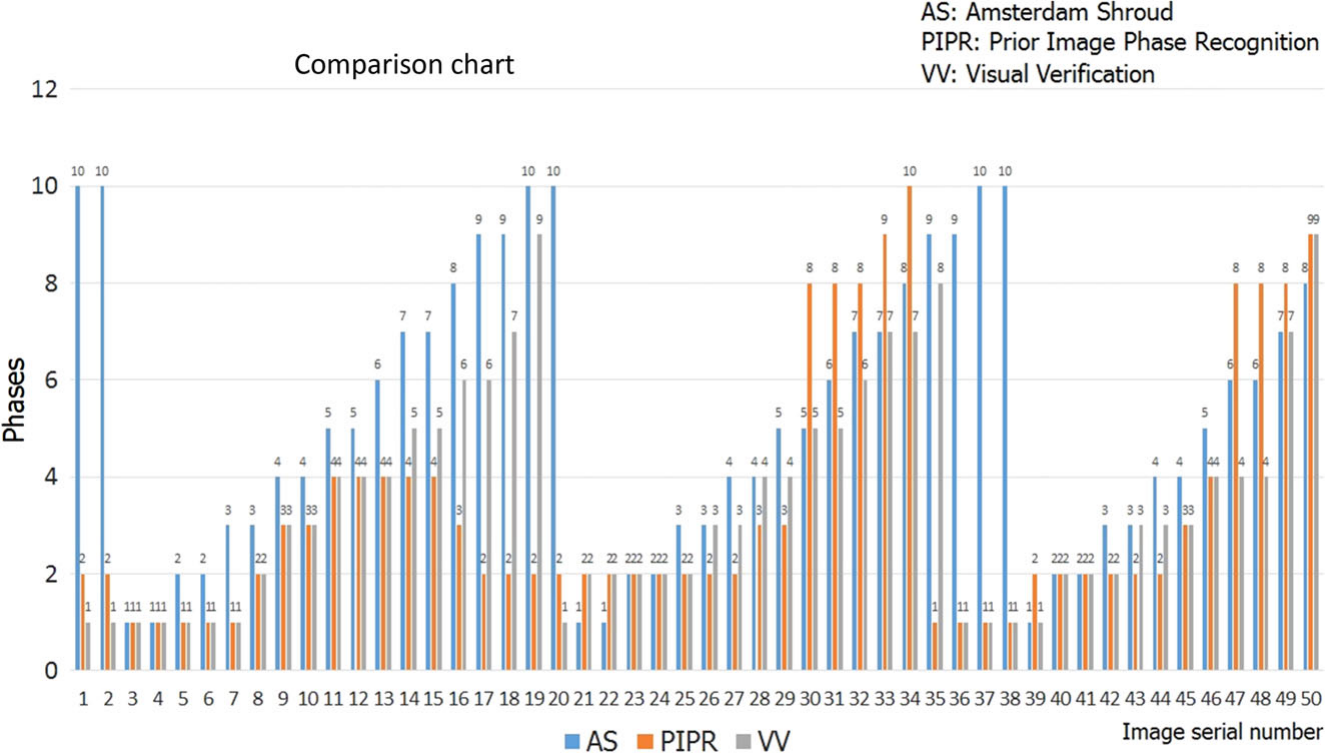
Comparison of AS and PIPR algorithm with visual verification for 50 tumor-visible images for the case of a patient treated with FFF beams.

Figure 6 shows the comparison chart for the first 50 images at a tumor-viewable angle and their respective phases obtained by AS, PIPR and visual verification for one patient.

**Fig. 7.**
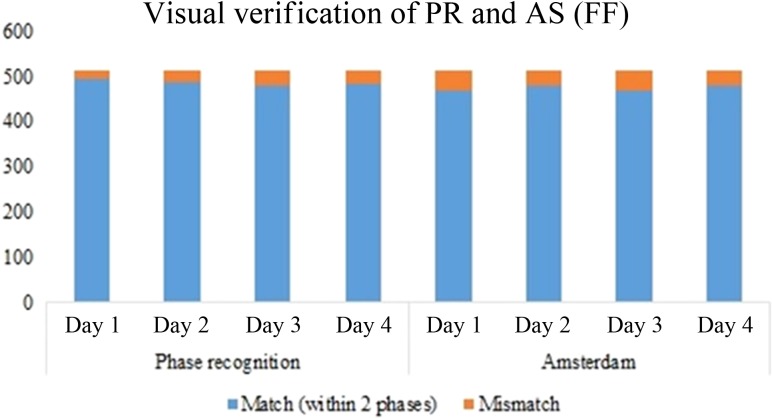
Verification of the AS and PIPR algorithm after visual phase recognition for 4 days for patients treated with FF beams.

Similarly, we performed the verification for all 20 sets of data, and the results for patients treated with the conventional FF beam for 4 days are illustrated in Fig. 7.

**Fig. 8.**
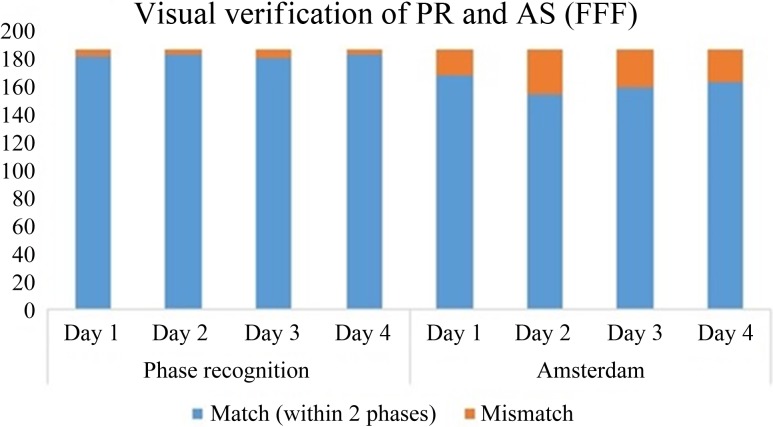
Verification of the AS and PIPR algorithm after visual phase recognition for 4 days for patients treated with FFF beams.

Similarly, the results for patients treated with FFF beams for 4 days are illustrated in Fig. 8.

The results indicated 97.5% accuracy using the visual verification in the case of patients treated with the FFF beam with phase recognition, and 83.5% accuracy when using the AS technique, whereas they indicated 95% and 93% accuracy, respectively, for patients treated with the FF beam. Phase differences within two phases were taken as indicating a correct phase, as described in Equation 3. The relative phase criterion described in the Materials and Methods section was also followed for visual verification. For example, if the results of cross-correlation for a few sequences were ‘3 4 4 5 5 6’, ‘3 4 4 3 5 6’, ‘3 4 4 4 5 6’, ‘3 4 4 5 5 6’, 3 4 4 6 5 6’ and ‘3 4 4 8 5 6’, then for the first five sequences they were considered to match the two-phase criterion. However, in the last sequence, phase 8 suddenly appeared; hence, we visually verified it to check whether it was closer to the fifth or fourth phase. Since respiration is mostly cyclic, a tumor in mid-exhale and mid-inhale phases has almost the same shape and position and might be expected to show a great similarity in its cross-correlation coefficient, with the values differing by ≤0.01. Hence, we need to use a relative phase criterion so that if the phase is ‘3 4 4 (7 or 8) 5 6’, that seventh or eighth phase would be assumed to be the fourth or fifth phase. In other cases, where there was no chance that the tumor was in that particular phase, we would take it as a phase mismatch and consider it an error.

While visually verifying the accuracy of our tumor tracking system, it was noticed that in the case of the first patient treated with FFF beams, the tumor motion was small during peak exhale, but was considerable during other phases. Tumor tracking using phase recognition based on template matching tracks the tumor itself, rather than the diaphragm, as is done using the AS technique, so the results were better for all phases when using our algorithm.

## DISCUSSION

This study focused on the development of a PIPR algorithm, based on prior-obtained 4D-CBCT images without the use of any external or infrared markers. Some recent studies have been reporting markerless tracking based on template matching [16, 17]. These studies were not based on any prior information, or in some cases were based upon planning CT images obtained days before the treatment. Phase recognition using 4D-CBCT obtained just prior to the treatment, with the patient in same position, provides a better knowledge of the patient’s breathing pattern and tumor motion, which is not believed to change drastically during the actual treatment session. For the five-patient cases used in this study, no significant baseline drift nor any drastic change was observed. In the case of large amplitude change between the prior 4D-CBCT data and that of the in-treatment session, our phase recognition algorithm must be used along with some other adaptive strategy through which a patient-specific phase margin can be created. The most important benefits of this system are: (i) this is a markerless tracking system; (ii) this system uses resources available in the hospital, i.e. without using any additional equipment or placing any extra burden on the patient compared with the current treatment process; (iii) this system uses the information obtained just before the treatment (the prior 4D-CBCT, i.e. 4D-CBCT acquired during the treatment positioning time); and (iv) this system is practical in terms of the requirements from radiation physicists and oncologists in the hospital.

In this study, we focused on phase recognition that is based on tumor matching. Increase in the number of the phase should not affect our results greatly, as we are matching the position of the tumor itself. The template phase images were prepared for all angles from +180° to –180°. On the other hand, the in-treatment images generally were limited to ~210 angles. This limit to the number of angles was a huge challenge in the verification study. Especially during a rotational treatment, the tumor was temporarily masked by high-density structures such as bone and spinal cord. Since we performed visual verification, we did not take the diaphragm position as the surrogate, as is the case with the AS technique. Due to this, our verification was limited to the number of images within the angles at which the tumor was visible, from approximately –135° to –67°. Of course, this will not limit our algorithm in future, because the additional information obtained from MV portal images will be useful in compensating for the limited number of angles. However, in this study, non-tumor-visible angles were only compared with AS phases.

Initially, the quality of the in-treatment kV images was a major concern in the case of the FFF beams due to the scatter photons from the MV beams and the rapid rotation in comparison with the FF beams in the VMAT treatment. However, the results in the case of FFF treatment beams indicate that there was not much distortion of the kV images compared with those obtained using the FF treatment beams. The percentage error in the visual verification was slightly higher in the case of FF patients. This was presumably due to the larger number of images for visual verification compared with the number for visual verification for FFF. However, in both FF and FFF therapies, the accuracy of verification using visual verification was higher than that obtained using the AS technique, because our algorithm tracked the tumor directly.

We have been working offline; hence, the AS phase data was already available. Also, real-time respiratory signal could be acquired by the Anzai-belt system (Anzai Co.) or other phase recognition method used in the hospital. However, a connecting algorithm to directly determine the phase based on images and the displacement of the tumor should be developed for working online. In this context, the Elekta response kit will allow us to control the beam delivery following a motion detection algorithm such as our PIPR algorithm. In a case for which our algorithm shows that the tumor is outside the two-phase criterion, the beam could be controlled using the response kit. The results obtained give the potential for developing a dynamic tracking system that can be applied in real-time with the MV beam-on for the tracking of moving tumors.

This study focused on assessing the ability to track tumors using phase recognition rather than the computing time. Though 100 ms on average for phase recognition would be sufficient for tracking in real time, the computational power could be increased by using a graphics processing unit (GPU)-based computer.

We aimed to develop a phase recognition algorithm (PIPR) using 4D-CBCT images acquired prior to the treatment. The re-projected data (template-projected images) were pre-prepared and then matched with the in-treatment projection images taken at the same angles. We also performed a comparison with the AS technique with visual verification. The result was an approximately 95–97.5% match in phase in all cases using phase recognition—an improvement on the 83.5–93% match obtained using the AS technique. The reason for the 2.5–5% error, in which the visually verified actual phase and obtained phase were three or more phases apart, could be some unusual breathing motion or some misjudgment during visual verification, which could be overcome by using some quantitative verification techniques in the future.

## CONCLUSION

Phase recognition based on 4D-CBCT has been successfully completed without the use of any external or internal markers. With successful phase recognition in FFF mode, several dosimetric benefits from FFF beams and tumor tracking are believed to be significant in lung tumor IGRT.
